# Intensity of Stripping and Sugar Content in the Bark and the Bast of European Beech (*Fagus Sylvatica*)

**DOI:** 10.1515/biol-2019-0003

**Published:** 2019-03-20

**Authors:** Tomasz Kurek, Jacek Todys, Witold Pazdrowski, Marek Szymański, Adrian Łukowski

**Affiliations:** 1 Polanów Forest Division, 12a Żwirowa Street, 76-010 Polanów, Poland; 2 University of Life Sciences in Poznań, Forest Utilization Department, Poznań Poland; 3 University of Life Sciences in Poznań, Department of Silviculture, Poznań Poland ,Institute of Dendrology PAS, Laboratory of Ecology

**Keywords:** *Cervus elaphus*, large herbivore, Polanów Forest District, red deer, tree stands

## Abstract

The choice of particular trees for stripping by deer is puzzling because a preference is observed for trees of the highest social class. Trees ranked highest in the stand can produce more carbohydrates as a product of carbon assimilation. Among the many important nutrient and defense groups of metabolites in plants, high sugar content is postulated to be an attractant due to its impact on the sense of taste of different herbivores. Thus, we hypothesize that the carbohydrate content in the bast of the European beech is the most important factor by which deer make their choice. Our aim was to assess whether the sugar content in the bark and in the bast can be a factor which influences the intensity of bark stripping of particular trees in a beech thicket by red deer. A single episode connected with tree damaging pertained to 7% of the trees on the analyzed sampling plots. In total, 27% of the trees were damaged, including old damages. The mean size of damage to a given tree equaled 36% of the tree’s circumference and 0.06 m^2^ of the trunk’s surface. Analysis of the sugar content in the bast and in the bark jointly indicates that the trees subjected to bark stripping possessed a distinctly higher sugar content than the trees which were not damaged. The probability of bark stripping grows together with an increase in the sugar content of the bark and of the bast.

## 1 Introduction

From the point of view of managing the population of ungulates and running forest husbandry, an extremely significant phenomenon is the influence of the ungulates on forest ecosystems and on the environment [1, 2, 3]. Large herbivore mammals can contribute to the following: the lowering of productive capabilities of tree stands [4]; the elimination of some species of woody plants [5]; and even the withering of trees as well as a complete change of vegetation cover [6]. On the other hand, deer (*Cervidae*) can contribute to a rapid settlement of new biotopes [7] and increase the productivity of habitats [2, 3]. To be more specific, deer can contribute by spreading plant and shrub seeds which are contained in their droppings. Furthermore, the growth of the population of large ungulates in Europe caused a revival of the population of large predators such as the gray wolf (*Canis lupus* L.) [8]. Damages caused by European deer (*Cervus elaphus* L.), consisting of bark stripping, browsing, deer rub, or breaking trees, gain importance both in a natural and in an economical way. Problems appear together with the damages caused by wildlife, and their consequences are becoming more and more severe. There recently has been noted a systematic growth of damages to tree stands caused by the deer not only in Poland but in all of Europe, as well [9]. Damages in the range of 21% - 41% of the area were ascertained in damaged tree stands in an area of 63,617 ha in Poland in 2015. And in the range above 40% - 26,610 ha [10]. The state-owned State Forests National Forest Holding bears the high expense of securing forests (of all ages) against damage each year. However, the effects do not meet expectations. The causes for this state of affairs are complicated. The growth in the number of the deer undoubtedly influences this as well as the limiting of their living space due to the fencing of tree stands and of arable farming. The most important species which damages tree stands in Poland through bark stripping is the red deer (*C. elaphus* L.) as well as the elk (*Alces alces* L.) which has recently gained an increasing economic importance. Furthermore, the European roe deer (*Capreolus capreolus* L.) is a sedentary species which can contribute in a significant manner to the limiting of the growth of crops by browsing valuable admixtures, among others. Moreover, cases of bark stripping by the forest horse present in Poland and by the European bison were also noted [11]. The red deer is a species which is present in 27 countries in Europe. It can also be considered to be the most important species of cervid which affects European forests [9]. The most important tree species in Poland from an economic point of view which are subject to bark stripping are the Scots pine (*Pinus sylvestris* L.), Norway Spruce (*Picea abies* [L.] Karst), as well as the European silver fir (*Abies alba* Mill.). There have also recently been noted cases of damages among broad-leaved species such as the European beech (*Fagus sylvatica* L.) and oak (*Quercus spp*. L.). While bark stripping of coniferous species is noted mainly in winter, beech is damaged only in the summer period.

**Fig. 1:**
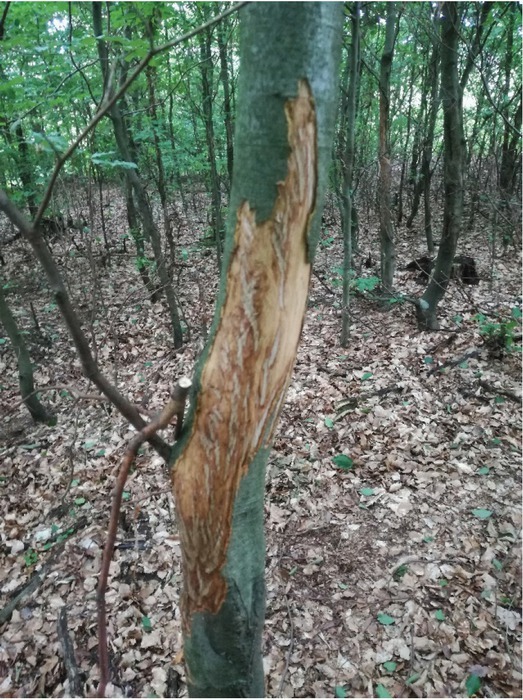
A few day old wound after bark stripping created in July

**Fig. 2:**
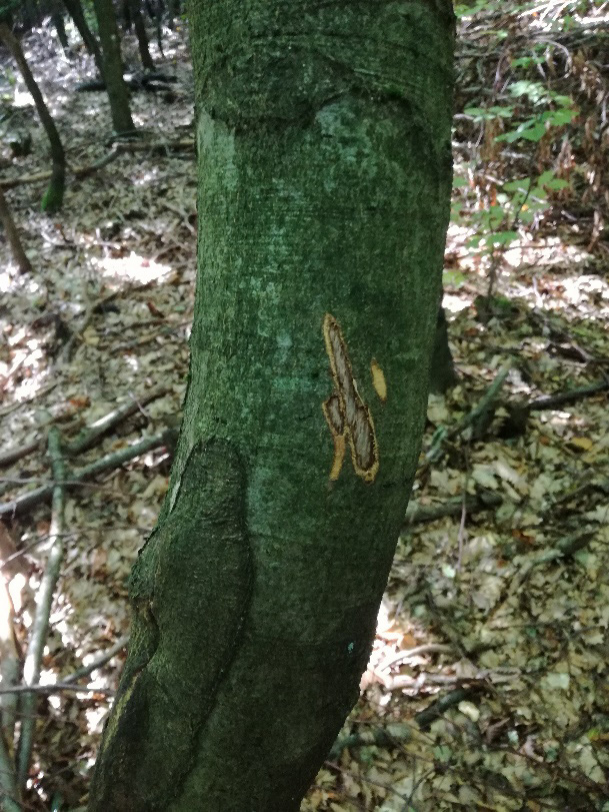
Tree that was “tasted” by the deer (*Cervus elaphus* L.)

Damages are present mainly during the vegetation period from May to August, when bark and bast are cooperatively easy to break away from the tree trunk (Fig. 1). We have observed in some cases that the individuals ,,tasted” the tree (Fig. 2).

**Fig. 3:**
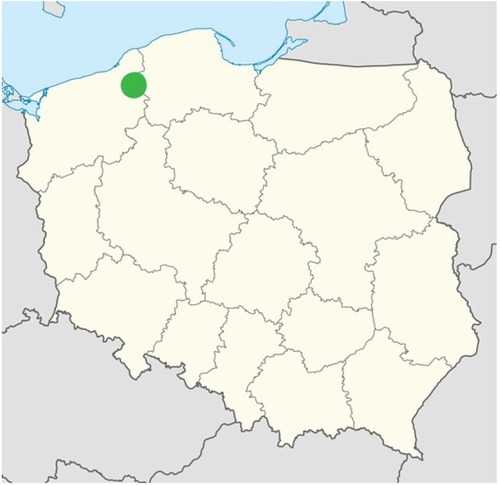
Localization of study area in Poland

In 2016 in the Polanów Forest District (Fig. 3), one of the thirty forest divisions which comprise the Regional Directorate of State Forests in Szczecinek (Poland), damages were inventoried in beech thickets in the range from 20% to 40% in an area of 124 hectares and in the range over 40% in an area of 37 hectares. In relation to 15 hectares of beech thicket, there are significant doubts in regard to the choice of proper future silviculture steps. The causes of such high pressure on tree stands are still difficult to determine. A team under the direction of Zbigniew Borowski, Ph.D., conducted a research project between 2013 and 2015. The aim of the project was to identify the causes of bark stripping of beech stands [12]. These authors point to the fact that summer bark stripping caused by the deer is not due to food shortage but is a search by the deer in the bark and in the bast for elements other than nutrients. Their results indicated the need for further research on specific substances, such as sugar, microelements, and fiber [12].

Previous observations and measurements conducted by the authors of this paper in the Polanów Forest Division territory indicate that the trees which underwent bark stripping belong to the highest social classes in Kraft Classification [13]. This influences further silviculture proceedings in such bark stripped stands in an obvious way. The trees with the highest diameter at breast height, which occupied the best social positions in the stand, were damaged to such an extent that it even caused them to wither. Such trees are extremely susceptible to breaking, insect damage, or fungal infections in subsequent years. As a consequence, this causes a complete change in the hitherto present spatial pattern of trees in the thicket [13]. The remaining trees belonging to the lower social classes, which previously fulfilled auxiliary functions, must assume the role of the primary component which constitutes the stand. The choice of particular trees by the deer is puzzling because, as it was previously mentioned, the observed pressure pertains to the trees of the highest social class. Simultaneously, there are noted cases of choosing secondary, dominated trees. This is interesting insofar as it can indicate the existence of a certain preference for choosing specific trees by the red deer. As the highest trees in the stand have their branches in the open and can produce more carbohydrates as a product of carbon assimilation. Among the many important nutrient and defense groups of metabolites in plants, high sugar content is postulated as an attractant due to its impact on the sense of taste of different herbivores [14, 15, 16]. Thus, we hypothesize that the carbohydrate content in the bast of the European beech is the most important factor by which deer make their choice. The aim of this paper was to assess whether the percentage of reducing sugar in the bark and in the bast can be a factor which influences the intensity of bark stripping of particular trees in the beech thicket by red deer.

## 2 Materials and methods

According to the forest classification, the Polanów Forest District is located in the I Baltic Region, the 5^th^ district of the Drawskie-Kashubian Lake District and the Drawskie–Pomeranian Lakeland (Fig. 3). The Polanów Forest Division is characterized by large moraine hills. A wildlife inventory was carried out in recent years by the means of the sample wildlife drive method. It indicated a relatively high number of red deer (*Cervus elaphus* L.). They equal roughly 90 deer per 1000 ha of forest area. The stand in the Żydowo Forest District in the 557d department (54°3’2,97N; 16°41’39,14E) was chosen for the research. The damages (bark stripping) were noted there in June 2016.

**Fig. 4:**
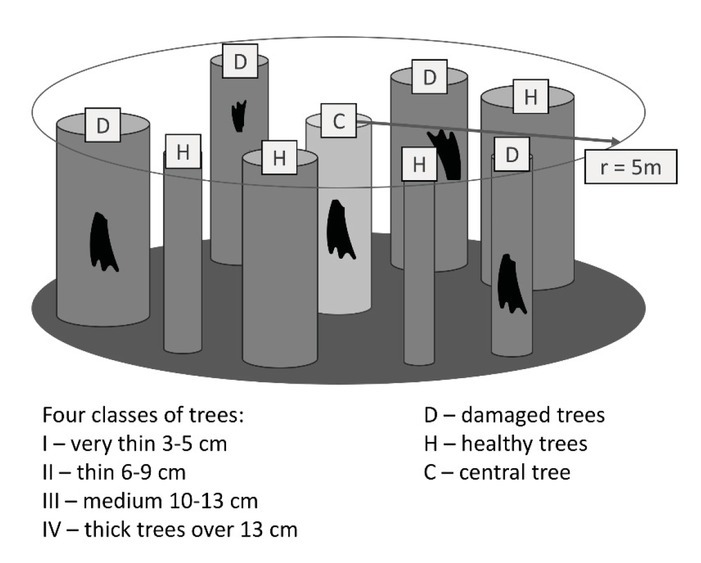
Research plot with four classes of trees

All 24 research plots were chosen in dense stands, and from these central trees were selected with stripped bark (C). Subsequently the trees were numbered and permanently marked with the use of tree marking paint. All of the trees within a radius of 5 meters from each central tree (C) were measured (Fig. 4). The measured characteristics included the following: the diameter at breast height and the present bark strippings, which were inventoried and their creation determined (this year’s – fresh, last year’s or older – old). The location of stripped bark on the tree was measured, from the current year as well as from previous years. The measurement of old and fresh wounds was necessary to determine how strongly the tree was wounded in a single episode of bark stripping. It is quite easy to distinguish fresh wounds because of the lack of regenerative marks (Fig. 1).

Research was divided into two steps, starting in June:

All of the bark strippings were measured and their length and width were determined together with their height from ground level. Roughly 300 grams of bark and bast were collected from the central trees (the damages ones) for the research. Samples were also collected from trees which were not damaged (referential); 6 samples were taken from each of the four ranges of the diameter at breast height: 3-6 cm; 7-9 cm; 10-13 cm, and over 13 cm. A total of 24 samples of bark and bast tissue were collected from the trees with stripped bark, and 24 samples were collected from the trees which were not damaged (healthy ones).

**Table 1:** Selected characteristics describing the researched tree stand

Department	Habitat	Tree stand type	Species composition	Quality class	Age	Silvicultural practices	Carried out treatment
557d	Coniferous fresh	Oak Beech	60% Beech	II	34	Commercial cut	2014
	forest		30% Beech	II	24		
			10% Oak	II	14		

Next step, in August

In August, samples were collected again in order to exclude or confirm a possible variation in the sugar content which could have been a consequence of the damages. The aforementioned samples were taken from twelve trees from the highest diameter ranges: 10-13 cm and over 13 cm. These trees had been previously marked as healthy (they served the role of referential trees in the course of the research), and they were damaged during the process of collecting samples for the research. It was impossible to collect for a second time material from the lowest diameter ranges (3-6 cm and 7-9 cm). The collected bast together with the bark were immediately transported to the Fertilizer and Chemical Products Laboratory of the Polish Center of Research and Certification Inc. in Warsaw, to the Pila branch. The samples were subjected to tests for total sugar content using the Luff-Schoorl titration method [17]. The most frequently used statistical tests were the nonparametric Kruskal-Wallis test and Spearman’s rank correlation. The vertical lines in the figures indicate standard error of the mean (±SE). The acquired results were subject to statistical analysis with use of MS Excel software as well as JMP.

## 3 Results

A total of 629 trees were measured in the course of the research. The mean diameter at breast height of the trees damaged due to bark stripping equaled 11.9 (±0.5) cm. And the in case of the trees which were not damaged (healthy), it equaled 6.4 (±0.4) cm. Also, healthy trees were inventoried in each sampling plot. These trees had been previously secured by applying special protective bandages which render it impossible for the deer to damage them. The mean diameter at breast height of such trees equaled 10.43 cm. The mean density of trees on the research area equaled 3,339 (±235) trees per hectare.

**Fig 5:**
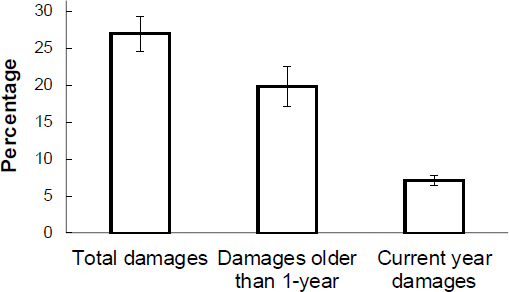
Percentage of damaged trees divided according to the period when the damage to the trees was made – this year or previously

The tree damages due to bark stripping were ascertained in almost 27 (±2.4) % of trees on the sampling plots (Fig. 5). From this percentage, approx. 20% of the trees had their bark stripped last year or earlier, and approx. 7% of the trees have been damaged during the current year. The length of bark strippings was in the spectrum from 89.2 cm to 148.9 cm (mean =118.9 ±4.7 cm). It was ascertained that the strippings which were located on the lowest level began 47 cm from the ground. The highest level reached by the bark stripping reached up to 173 cm. The mean circumference of damaged trees equaled 38.2 (±0.5) cm, while the largest one equaled 56.1 cm.

**Fig 6:**
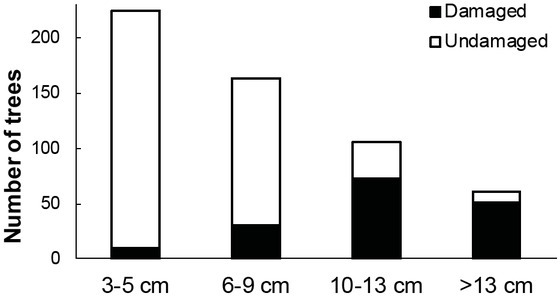
Breakdown of damaged trees according to the diameter class of the tree

The concentration of the damages was present in four highest diameter classes (Fig. 6). Approx. 84% of trees with a diameter at breast height level of 13 cm were damaged as well as 69% of trees with a diameter at breast height in the range of 10–13 cm. A percentage increase in damage in the stand was observed with the growth of the diameter at the breast height classes. Nonparametric Spearman’s ρ correlation coefficient illustrates the relation between the diameter at breast height and stripping area (ρ = 0.574; p = 0.003).

An analysis of the reducing sugar content in the bast and bark jointly indicates that the trees subjected to bark stripping possessed a distinctly higher sugar content (1.37 (±0.07) %), than the trees which were not damaged (0.43 (±0.04) %). This content is more than three times higher. A variation in the percentage of sugar in the samples collected from the referential trees (from which the research material was collected earlier) grew only slightly and insignificantly (χ^2^ = 2.28; p = 0.13) from 0.46 (±0.04) % in June to 0.56 (±0.07) % in August. At the same time, a significant decrease (χ^2^ = 7.05; p = 0.008) in the sugar content from 1.25 (±0.07) % to 0.92 (±0.11) % was noted by analyzing the trees with bark stripping. Pearson correlation coefficient illustrates the lack of relation between the tree’s trait and conditions (diameter at breast height, tree girth, stripping lengths, etc.) and the sugar content (low value of the coefficient and lack of significance).

It was observed that there are trees which were damaged again this year even though they had been already stripped in the past. For this reason, it was decided to compare the sugar content in these trees with the trees which were stripped for the first time this year. The results indicate that the trees without old bark stripping which were damaged in 2016 for the first time possessed the same amount of sugars in the bast and the bark as the trees which possessed old bark strippings on the side of the trunk (χ^2^ = 0.104; p = 0.747).

## 4 Discussion

The acquired results illustrate numerous interesting dependences. Furthermore, they shed a new light on certain issues connected with bark stripping of European beech stands by red deer. The high concentration of damages in the two highest diameter classes can cause significant problems with further silviculture of these stands because the trees which were subject to bark stripping constitute their fundamental framework. The answer to the question pertaining to the further silviculture of such tree stands remains without a doubt to be a huge challenge for forest managers. One of the alternatives that remains is securing trees individually (by different means). According to Borowski [12], the most effective method of securing beech trees involves using a chemical product (Cervacol). However, according to the authors of this work, long-term observations indicate that bandages seem to be the more effective solution. With the passage of years, a change in the vertical layout of trees occurs in the beech thickets. For this reason, the movement of trees from a higher social class to a lower class should also be taken into account during the planning stage in which the trees are selected for protection. For comparison with our results, the mean damages in the forest divisions researched by Rutkowski amounted to 20% in the Człopa Forest Division, 23% in the Leśny Dwór Forest Division, and 26.8% in the Czaplinek Forest Division [18]. In the next stage, an analysis of the sugar content from the referential trees (the ones from which samples were collected previously) was carried out. This was done in order to study whether high sugar content is not the result of an increase of sugar content in phloem juice as a reaction of the tree to the resultant damages. The results of the analysis did not demonstrate a significant increase of the sugar content due to mechanical damages. In the case of the 8 trees with bark stripping from which research material was collected again, a slight decrease in sugar content was observed. This can be explained by the fact that these trees went into the death phase because they practically completely underwent girdling. 12 out of the 24 analyzed trees with bark stripping (50%) possessed old bark stripping which was made in the previous year or earlier. Consequently, an additional comparison of the sugar content in the trees damaged in the past and of the trees damaged for the first time was carried out. The acquired results indicate that the trees without old bark stripping, damaged in 2016 for the first time, possessed the same amount of sugars in the bast and in the bark as the trees which possessed old bark strippings on the side of the trunk. Even though a different sugar content was found in the group of the examined damaged and healthy trees, the maximum and the minimum as well as the course are similar in the case of both healthy as well as damaged trees. This can point to the fact that the damaging of trees through bark stripping does not influence a change in the level of the sugar content in the phloem juice. If we assume that the trees with the aforementioned values of diameter at breast height ranges occupy similar social positions in the thicket, then one can put forward a thesis that the differences between the sugar content in the healthy and the damaged trees can result from variability between trees. Several factors can influence this, namely, changeability of the microhabitats, the size of the crown, or other unknown factors.

Numerous authors analyzed the chemical composition of the bark and of the bast as a factor which influence the choice the ungulates make between various tree species or between individual trees within a given species [11]. The results differed significantly and showed a lack of specificity [11, 19, 20] or showed certain differences in the intensity of bark stripping according to chemical composition [11, 21]. In the context of the rapid physiological reaction of trees to bark stripping [11, 22], it remains unclear whether the susceptibility to bark stripping depends on the chemical composition of the outer bark [11, 22, 23]. A tendency to separate the outer bark can be another factor which influences the difference in the intensity of bark stripping between tree species and among the individuals of a particular species. Theoretical framework for the physical aspects connected with the separation of the bark was provided by De Crombrugghe [11, 24]. The differences in the properties of the stripped tissue between the seasons were connected with changes in the structure of the tissue such as easier separation during the summer rainy season. This was in turn explained by a decreased elasticity of ground tissue (parenchyma) and faster lignification of cell walls during the dry season. This was indirectly confirmed by Saint-Andrieux et al. [11, 22] and his measurements of the length of the stripped bands. This shows a seasonal changeability in susceptibility to the separation of the bark and the bast in beech. The water content in the outer bark was also positively correlated with the frequency of bark stripping by red deer. This indicates that in spring and in summer a larger amount of the outer bark of beech trees was separated than in autumn and winter. Various tree species can possess a different level of water storage which, consequently, can influence the structure of the outer bark [25]. As a result, differences in the adhesive force of the outer bark can be observed between the species [11, 26, 27]. Even though other authors pointed to the role of physical properties at the time of the separation of the bark to explain the selectivity of deer in the selection of a tree for the purpose of bark stripping [11, 19, 23], there are no conclusive measurements hitherto to document this argument. The results of the research by Klich [11] indicate that the force necessary to separate the outer bark expressed in newtons (N) and the relative force (N/g*dm) is different among species. Furthermore, these differences are not always statistically significant. The force necessary to separate the outer bark decreased significantly together with the diameter at breast height. The trees analyzed in the Polanów Forest Division were damaged at different diameters at breast height ranges. However, the majority of the trees which were damaged had the highest diameter at breast height values. Only a small number of trees from the two lowest ranges of diameter in the 4-stage scale were damaged. To be more precise, only 4% and 18%, respectively, of the trees from those groups were damaged. In the analyzed case in the Polanów Forest Division, the argument about the preference for thinner trees can be excluded with a large dose of certainty.

Rajský et al. [28] show that the strong negative correlation between bark stripping and the energy that is available to attain can attest to the fact that the acquired metabolic energy is the main factor which influences the size of the bark stripping. The authors recommend that this should be taken into consideration in case of feeding if one wishes to decrease the amount of bark stripping. The results acquired in the Polanów Forest Division seem to confirm this principle to a certain degree. The analysis of the dependence between the sugar content in the bark as well as the bast of trees and the size of the bark stripping indicate that a dependence exists between the width and the length of the strippings as well as the percentage of sugar in the trees. The bark strippings were larger in the case of trees with less sugar. So, in order to achieve the same energy effect, deer required a little more food. Consequently, they could make larger bark strippings. One of the frequent issues connected with determining the reason behind bark stripping by deer is studying whether the animals have easy access to water. The bark and the bast of trees are comprised mainly of water [29], and the deer search for succulent food in summer. Young bark and bast which intensively carry assimilates in this period contain up to 60% of water. Researchers comparing the size of the bark stripping arrived at interesting conclusions. They compared bark strippings depending on whether deer were given silage (grass silage, corn silage) or dry, concentrated pellets. All of the animals had access to water in the course of the research. No differences were determined in the size of the damage. So it can be thought that water content in the food does not influence the intensity of bark stripping [28, 30]. Access to water is indispensable when the deer are given only dry food such as pellets. This information is extremely crucial in the context of possible supplementation of minerals in the form of pellets because damages in tree stands could increase without them. In the year when the research was conducted at the Polanów Forest Division, precipitation amounted to 403.6 mm in the period from March to July and was higher than the average (303.4 mm) recorded between 1987 and 2015 (data acquired from the Geoecological Station in Storkowo, which belongs to Adam Mickiewicz University in Poznan). Furthermore, there are numerous natural water reservoirs in the vicinity of the analyzed department which the animals can enjoy freely. Consequently, the argument about the necessity of water retention for red deer can also be excluded.

According to Saint-Andrieux et al. [22], bark-stripped trees had a slightly higher carbohydrate content than non-stripped trees, but this difference resulted from a physiological reaction of the tree to bark stripping. The difference observed in our study between stripped and non-stripped trees was higher than that of artificially stripped trees.

Jarnemo [31] presented interesting deliberations as well as a comprehensive review of literature pertaining to the reasons behind bark stripping of trees by deer. The author claims that the main reason behind the changes in the vegetation cover caused by deer is a high concentration of these animals [30, 31, 32, 33]. That is why the main measure designed to decrease the damage caused by these animals is decreasing the concentration of the population [34, 35, 36]. However, it is important to remember that the concentration of the deer can only be one of many factors which influences the intensity with which the damages occur. Also, the concentration itself can be dependent on other factors [31, 37 - 39]. In such cases, if the dependence between the concentration of the deer and the intensity of the damages is weak, reduction of the population may not bring about regeneration of the vegetation cover [31, 38, 38, 39, 40]. A relatively numerous population of red deer is present in the Regional Directorate of State Forests in Szczecinek. There were 26,892 specimens in 2016 in comparison to 12,831 specimens in 2002. There was a mean gain in numbers from 7 specimens per 1000 hectares (the Osusznica Forest Division) to 34 specimens per 1000 hectares (the Czaplinek Forest Division) in the past four years. The mean change in deer numbers equaled 20 specimens per 1000 hectares of forest area in the Regional Directorate of State Forests in Szczecinek. After the comparison, there is no visible correlation between the values of the size of the deer population with the damages inventoried by the forest service in the beech tree stands in the forest divisions of the Regional Directorate of State Forests in Szczecinek. For instance, there is a mean of 20 specimens of deer gained per 1000 hectares of forest area in the Leśny Dwór Forest Division, and there were almost 400 hectares of inventoried beech thickets which were damaged in an area of over 20%. In contrast, there were only 28 hectares inventoried of such tree stands in the Połczyn Forest Division where 27 deer were gained per 1000 hectares. The reasons behind this state of affairs could be a number of factors. One of these is the methodology itself of inventorying such damages, which can cause numerous problems, especially in tree stands which were created through natural regeneration. Another reason can be the length of occurrence of this phenomenon in a particular area as well as the hitherto undertaken protective action. An obvious potential factor which influences the size of the damage in the tree stands is the species and age structure of the tree stands as well as the structure of arable farming which neighbors the forests. The next topic discussed by the researchers is the issue of disturbing the animals and the stress associated with, among others, hunting or scaring the wildlife away. The phenomenon of bark stripping under heavy stress has the characteristics of a nervous tic. It functions as a form of transference of cumulative stress [41]. Researchers who compared bark stripping on laid down trees by two groups of deer came to interesting conclusions. The deer which were stripping the bark were kept in farming conditions, and one group was subjected to disturbances which simulated a hunt in progress. Access to food for animals was limited from 7:00 a.m. to 5:00 p.m. in this period and during the hours which were supposed to resemble conditions present in places where hunts are carried out. Consumption of the bark and the bast increased significantly in this group. In the group of deer which were disturbed and which were fed grass silage, the consumption of fibrous sustenance grew from 19 g daily to 470 g daily. And, in case of corn silage, consumption grew from 6 g daily to 101 g daily [28]. The authors claim that the degree of bark stripping increases as the animals get more and more disturbed. Moreover, in order to limit the damage, they think that the optimal form of feeding is laying out feed comprised of hay mixed with corn silage. In Poland, the most intensive period of disturbing wild game takes place from the 21^st^ of September until the end February, which is when deer season is open. Additionally, between July and the end of October, pickers of vegetal cover (berries, mushrooms) are frequently present in the forests, and they also are a source of disturbance in the hunting ground. However, the greatest disturbance in Polish forests takes place in the period when the stags shed their antlers (end of February-March). This is also the time when many people penetrate forest areas in order to acquire antler sheddings, which they later sell for profit. In the period of emergence of the damages in the beech tree stands (June-July), an intensive forest penetration by people is not noted. This is a period of relative peace, so the aspect of scaring animals and the stress connected with this should also rather be excluded. In the course of the research carried out in June 2016 in the Polanów Forest Division, exceptionally interesting results were acquired which confirm that deer search for specific nutrients. High sugar content in 71% directed the occurrence of damages in the beech tree stands. There still remains an open question about whether the deer want to acquire high-energy food this way or if this is only a supplement complementing their diet. Another extremely intriguing issue pertains to the period of creating damages because this occurrence intensifies in the summer period (June-July). This is a time when there is no problem with access to food, for example, on the fields and meadows. On the other hand, it should be remembered that beech tree stands, which are the natural habitat of deer, offer a very poor food base. According to Rutkowski [18], there are only 2 to 14 herbaceous plants present in the coniferous fresh forest and mixed fresh forest habitats. High stand density results in a mostly bare vegetal cover in the lower story. As a consequence, the bast and the bark of trees can be an attractive substitute for food which is simply not available. Can a heightened need for nutrients be connected with the fact of approaching rut and the need to prepare the organism for procreation? The answer to this question may change how we approach the management of red deer population in Poland. So feeding and supplementing nutrients will become an auxiliary tool in limiting damages to the tree stands instead of causing ,an excessive concentration of animals in one area which generally leads to an increase of the damages in the stands. The results of the hitherto conducted research demonstrate the necessity to continue these studies in order to delineate factors which determine the creation of damages. This will enable confirmation on a larger sample with the help of statistical methods on the basis of the previous results. An applicative effect should also be the development of protection and husbandry procedures. These will also pinpoint how to prevent the occurrence of damages in the future (with the use of supplementation of the deer diet) as well as how to deal with the already damaged stands.

## 5 Conclusions

A single episode connected with tree damaging pertained to 7% of the trees on the analyzed sampling plots. In total, 27% of the trees were damaged, including old damages.

The mean size of the damaged band measured at the circumference of the tree equaled 36% of the tree’s circumference.

The ascertained damages belonged to a significant group a mean size of 0.06 m^2^ of a trunk’s surface was damaged.

69% of the trees from the diameter range of 10–13 cm were damaged. And in the diameter range of over 13 cm, 84% of the trees were damaged.

The probability of bark stripping grows together with the increase of the sugar content in the bark and in the bast.

The results of the present measurements and observations allow us to ascertain that most probably the determining factor of the so-called summer bark stripping of beech as well as the selection of particular trees by deer is the presence of specific elements in the bark and in the bast of the trees.

The level of the sugar content in the bark and in the bast of the trees was measured by the means of using the Luff-Schoorl titration method and they differ quite significantly between the damaged and the undamaged trees.

The analysis of the collected results connected with the concentration of sugar in the bark and in the bast of the beech trees which were subjected to bark stripping or were not subjected to bark stripping by deer in different time periods and the ones which were damaged artificially indicate that the damaging of a tree does not exert an influence on the increase of the sugar concentration.
